# Light-trapping and recycling for extraordinary power conversion in ultra-thin gallium-arsenide solar cells

**DOI:** 10.1038/srep28303

**Published:** 2016-06-23

**Authors:** Sergey Eyderman, Sajeev John

**Affiliations:** 1 Department of Physics, University of Toronto, 60 St. George Street, Toronto, Ontario, M5S 1A7, Canada

## Abstract

We demonstrate nearly 30% power conversion efficiency in ultra-thin (~200 nm) gallium arsenide photonic crystal solar cells by numerical solution of the coupled electromagnetic Maxwell and semiconductor drift-diffusion equations. Our architecture enables wave-interference-induced solar light trapping in the wavelength range from 300–865 nm, leading to absorption of almost 90% of incoming sunlight. Our optimized design for 200 nm equivalent bulk thickness of GaAs, is a square-lattice, slanted conical-pore photonic crystal (lattice constant 550 nm, pore diameter 600 nm, and pore depth 290 nm), passivated with AlGaAs, deposited on a silver back-reflector, with ITO upper contact and encapsulated with SiO_2_. Our model includes both radiative and non-radiative recombination of photo-generated charge carriers. When all light from radiative recombination is assumed to escape the structure, a maximum achievable photocurrent density (MAPD) of 27.6 mA/cm^2^ is obtained from normally incident AM 1.5 sunlight. For a surface non-radiative recombination velocity of 10^3^ cm/s, this corresponds to a solar power conversion efficiency of 28.3%. When all light from radiative recombination is trapped and reabsorbed (complete photon recycling) the power conversion efficiency increases to 29%. If the surface recombination velocity is reduced to 10 cm/sec, photon recycling is much more effective and the power conversion efficiency reaches 30.6%.

Silicon has been a material of choice for direct solar to electrical power conversion due to its abundance, reliability and mature manufacturing technology. However, the indirect electronic band gap of silicon hinders absorption of sunlight at long wavelengths. Gallium Arsenide (GaAs), on the other hand, has a direct electronic band gap and can provide greater power conversion efficiency. Since it is more scarce and costly than silicon, it is vital to optimize solar absorption using the least amount of GaAs. This can be achieved by sculpting GaAs into sub-wavelength periodic nanostructures known as photonic crystals. In this work, we show using coupled numerical modeling, that nearly 90% of sunlight, in the available wavelength range, can be trapped and absorbed in ultra-thin films of nanostructured GaAs consisting of only 200 nm of GaAs. This enables photonic crystal solar cells, utilizing less than one-tenth the volume of GaAs than state-of-the-art single-junction GaAs solar cells, to achieve record high power conversion efficiencies, beyond 30%.

Resonant light scattering and wave-interference effects provide powerful light-trapping mechanisms^1^,^2^ in nanostructured thin-film solar cells. It has been shown^3^,^4^ that these wave effects can be exploited in certain photonic crystal^5^,^6^,^7^,^8^,^9^,^10^ designs to achieve very high solar absorption using one or two orders of magnitude less active material than conventional solar cells. These photonic crystal architectures have an effective graded refractive index near the top surface to minimize reflection and scatter incoming sun light into slow-light modes that propagate nearly parallel to the interface between the active region and air above^5^. The resulting long dwell time of sunlight within a thin-film enables stronger light absorption than anticipated in the so – called Lambertian limit^7^. Enhanced solar absorption occurs in a wavelength range in which the electromagnetic density-of-states is much higher in the photonic crystal than in a homogeneous medium. These effects cannot be described using a ray optics picture and require detailed numerical solution of Maxwell’s wave equation within the solar cell. In the case of relatively thick (~100 microns) silicon solar cells, a detailed balance method^11^ based on Lambertian ray trapping has been used to estimate the limiting power conversion efficiency. However, this approach neglects wave interference effects that can provide stronger light-trapping and solar absorption using considerably thinner photonic crystal solar cells^7^,^12^.

The maximum Shockley-Queisser^13^ solar power conversion efficiency for a single junction GaAs solar cell is around 33.5% using an AM 1.5 solar spectrum. Recently, the efficiency of ~3 micron thick single-junction GaAs solar cells has improved considerably, reaching almost 29%^14^,^15^,^16^. By minimizing surface non-radiative recombination and maximizing photon trapping^8^ and recycling effects^17^,^18^,^19^,^20^,^21^,^22^,^23^ further efficiency increase toward the Shockley-Queisser limit remains possible.

An order of magnitude reduction in the volume of GaAs required to achieve such high efficiency is an equally important target. A two-fold strategy is essential to realize these goals. Firstly, optimized light management requires simultaneous antireflection and light-trapping^5^,^6^,^7^,^8^. This enables higher photon collection efficiency and correspondingly higher photo-current. Light-trapping also facilitates re-absorption of photons generated by electron-hole recombination. This effect, known as photon recycling^19^,^20^,^21^, leads to further charge carrier generation and an increase in open-circuit voltage. Under suitable circumstances, as we show below, this provides up to 2% additive increase in overall power conversion efficiency using only 200 nanometers equivalent bulk thickness of GaAs (similar additive increase was found using active layer thicknesses of more than a micron^22^,^23^).

Secondly, optimized electronic management involves minimization of Schottky-Read-Hall^24^ and other non – radiative recombination. Non-radiative carrier recombination is a particularly critical issue in the case of large surface and contact areas. To prevent large losses, surface passivation^25^ is required. Unpassivated surfaces, defective with dangling bonds, induce rapid non-radiative recombination. This is a major factor in the low open-circuit voltages observed in some fabricated solar cells^26^. Recent advances in surface passivation facilitate the deployment of nanostructured solar cells with a large surface area. For example, surface recombination velocities^27^ as low as 10 cm/s have been achieved on black silicon, using an aluminum oxide coating. The concentration of defects is usually maximal at metallic contacts. Metallic back-reflectors serving as a back contact generally need to be offset from the active region of the solar cell to reduce losses. Building electronic barriers also helps to deflect minority carriers away from the points where electrical contacts touch the active region. Such heavily doped regions function through what is called a back-surface-field^24^. Without proper minority carrier deflectors, the recombination loss at contacts can be significant. Substantial increase in solar cell performance is achieved by addressing these photonic and electronic management issues.

In this paper, we present the results of combined numerical solution of Maxwell’s equations coupled to semiconductor drift diffusion equations in ultra-thin-film gallium arsenide photonic crystal solar cells. With only 200 nanometers equivalent bulk thickness of GaAs, it is possible to achieve solar power conversion efficiencies near 30%. We show (Supplementary Information) that this choice of thickness provides the optimum balance between high power conversion efficiency and the volume of GaAs required. We also highlight the interplay between carrier radiative recombination leading to “photon recycling”, and surface non-radiative recombination. In particular, a well-passivated surface can lead to nearly 2% additive increase in power conversion efficiency through the trapping and recycling of internally emitted light.

We present full 3D numerical simulations of photo-current, voltage and power conversion efficiencies in thin-film photonic crystal solar cells based on slanted conical nano-pores in GaAs^8^. Preliminary slanted-conical-pore structures have been fabricated on small scales using ion beam etching at S. Juodkazis group (private communication) and closely related vertical-pore structures have been fabricated on large scales using dry-etching methods at S.Y. Lin group (private communication). Light-trapping in a slanted conical nano-pore architecture enables 90% solar absorption in the wavelength range from 300–860 nm using only 200 nm equivalent bulk thickness of GaAs^8^. We calculate the dependence of power conversion efficiency on the degree of surface passivation. Despite the significant surface profile of our photonic crystal, efficiency is high provided the surface recombination velocity remains below 10^4^ cm/s. We also show the increased role of photon recycling when the surface recombination velocity is reduced by improved passivation.

While untreated GaAs can have an extremely high surface recombination velocity (10^6^ cm/s), passivation with a wide band gap semiconductor such as Al_x_Ga_1−x_As provides long-term surface stability. For epitaxially- passivated, planar GaAs surfaces, recombination velocities below 60 cm/s have been reported^28^. Nonplanar GaAs surfaces are harder to passivate, because of a higher density of electronic surface states. For GaAs nanowires^29^, a surface recombination velocity of 1700 cm/s was reported, indicating that the surface states were only partially passivated or that stacking faults and bulk impurities were further contributing to carrier non-radiative recombination. Advances in epitaxial growth of III-V semiconductors through complex 3-D structures with non-planar surfaces are nevertheless forthcoming^30^. In the present study, we demonstrate a strong synergy between surface passivation and photon recycling effects, highlighting the efficacy of surface recombination velocities as low as 10 cm/s. Although this lowest value has not yet been experimentally achieved for GaAs, we anticipate that our theoretical roadmap may stimulate research in this important direction.

To calculate an efficiency of our photonic crystal solar cells, we combine optical and electrical finite-difference time-domain (FDTD) simulations^31^. The solution of Maxwell’s equations provides the absorption profile inside the structure that defines the charge carrier generation profile. This profile is used as the input for the semiconductor drift-diffusion equations to calculate solar cell efficiency, taking into account radiative recombination throughout the bulk, and non-radiative recombination at surfaces. When all internally generated light from radiative recombination is assumed to escape the solar cell into free space, we obtain a power conversion efficiency of 28.3% in our 200 nm GaAs photonic crystal with surface recombination velocity of 10^3^ cm/s. In the opposite limit, when all light from radiative recombination is assumed to be recycled, this efficiency improves to 29%. Most strikingly, if the surface recombination velocity is reduced to 10 cm/s, the role of photon recycling is significantly elevated. In this case, complete recycling of re-radiated light leads to a theoretical power conversion efficiency of 30.6%.

## Numerical model

We calculate solar absorption inside our solar cell using the standard FDTD algorithm^32^,^33^, in which a plane wave impulse having Berenger’s form^32^ with a broad spectrum impinges onto the structure. Electromagnetic fields are recorded, transformed to the frequency domain and normalized to the incident spectrum. We define (in CGS units) a frequency – and spatially – dependent absorption coefficient at each point *r* inside the structure:


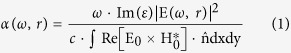


here, ω is the angular frequency, 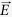 is the electric field amplitude calculated at each point of a computational grid located within GaAs, *ε*(*ω*) is the frequency-dependent and complex dielectric permittivity of GaAs^8^, c is speed of light, *E*_0_ and *H*_0_ are electric and magnetic vectors of the incident plane wave, the superscript * indicates the complex conjugate, 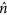 is a normal unit vector pointing from air to the surface of the absorbing medium, and integration is over a unit cell area of the surface. If 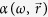 is integrated over all 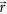 in the unit cell volume (defined by the unit cell surface area times the depth of the absorbing medium), it yields the frequency - dependent absorption coefficient: 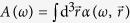. Assuming that each absorbed photon leads to the generation of a single e-h pair, we calculate an initial charge carrier generation rate (in units of number per unit time, per unit volume) produced by the incident sunlight. This generation rate is obtained by the integration of the calculated absorption 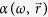 with the incident Air Mass Global Spectrum intensity *I*(*ω*) over the wavelength range 300–865 nm:


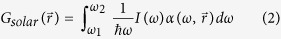


We use this spatially – dependent generation rate as an input parameter to the semiconductor drift – diffusion equations:


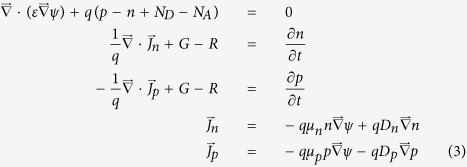


Here, R and G are the total recombination and generation rates, p and n are electron and hole densities, N_A_ and N_D_ are acceptor and donor doping concentrations, ψ is the electrostatic potential, q is an elementary charge, ɛ is the dielectric function of GaAs, J_n_ and J_p_ are electron and hole currents and μ_n_ and μ_p_ are electron and hole mobilities. In principle, the total generation rate G = G_solar_ + G_recycle,_ where G_recycle_ is the generation rate from re-absorbed photons produced internally by electron-hole recombination. The electron-hole recombination rate here is a sum of contributions from radiative and non-radiative processes: R = R_rad_ + R_SRH_, where R_SRH_ is the Shockley-Read-Hall non-radiative recombination from a single-trap level at the electronic midgap^24^ of GaAs. In our ultra-thin film photonic crystal, we can safely neglect bulk non-radiative recombination (R_SRH_ = 0), since the diffusion length of crystalline GaAs is more than one hundred times the maximum charge carrier transport distance in our structure. The non-radiative recombination consists entirely of surface recombination, which is treated as boundary condition. This is prominent at GaAs surfaces, especially near electrical contacts. Non-radiative recombination is described by the boundary conditions: 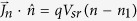, 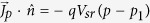, where 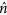 is the unit normal vector to the surface, V_sr_ is a surface recombination velocity and n_1_, p_1_ are equilibrium electron and hole concentrations defined below. The boundary condition for the electrostatic potential at the metal-semiconductor interface is: *ψ* = *V*_*appl*_ + *ψ*_*bi*_, where *V*_*appl*_ is the voltage applied across the contacts (we put V = 0 at upper contact and then *V*_*appl*_ at bottom contact), *ψ*_*bi*_ is the built-in potential (potential across the depletion region in thermal equilibrium that inhibits further carrier diffusion across the junction) given by: 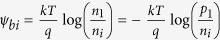, where T is temperature and n_i_ is the intrinsic carrier concentration of GaAs (*n*_*i*_ = 1.9·10^6^ cm^−3^ at T = 300 K). Equilibrium concentrations are given by^24^:


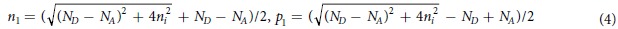


These concentrations are simple consequences of the charge neutrality condition: *N*_*D*_ + *p* = *N*_*A*_ + *n* and the law of mass action: 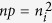.

Radiative recombination of electrons and holes (in units of number per unit time per unit volume) is given by^24^:


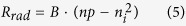


here, *B* = 1.3·10^−10^ (with units of volume per time) is the recombination coefficient^24^ of GaAs.

For the semiconductor-insulator interfaces we use following Neumann boundary conditions: 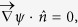 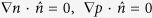, which implies that there are no surface charges (the normal component of the electric field is zero) and no current flow through the surface.

We discretize (3) using the Scharfetter–Gummel scheme^31^. This leads to a system of nonlinear equations that can be efficiently solved by Newton’s technique^31^. In general, photon recycling requires repetitive iterative solution of the set of equation (3) in steady state. While the solar generation rate G_solar_ is known, the internal generation rate G_recycle_ is an outcome of calculation. In principle, G_recycle_ is obtained by placing FDTD sources that emit with an intensity governed by the rate R_rad_ in equation (5) and then calculating absorption from these sources, using Maxwell’s equations throughout the active region. This process must be repeated until the input generation rate and the output generation rate converge throughout the sample volume. This iterative process will be described in detail elsewhere. For simple estimation purposes, we consider two limiting cases. In the first case, we assume that all light from radiative recombination simply escapes the solar cell without re-absorption. In the second case, we assume that all light from radiative recombination is trapped by the photonic crystal architecture and re-absorbed very close to the emitter. The latter is implemented by setting G_recycle_ = R_rad_ throughout the sample. As we discuss below, our light-trapping photonic crystal architecture enables re-absorption of nearly 70% of the re-emitted light.

## Solar Cell Architecture

**Figure 1:**
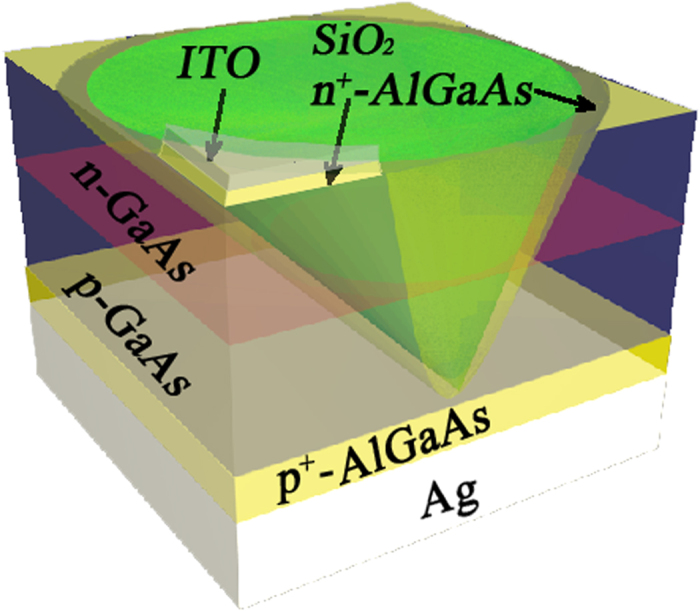
Unit cell of square lattice photonic crystal solar cell architecture based on slanted conical pores in bulk GaAs filled with glass. The photonic crystal slab rests on a silver substrate of 100 nm width. The depth of GaAs cones is 290 nm (for a 200 nm equivalent bulk thickness of GaAs). The radius of cones and the lattice constant are r = 300 nm and a = 550 nm respectively. The ITO 10 nm thick contact (refractive index n = 1.9) rests on 10 nm thick Al_0.4_Ga_0.6_As with higher doping (N^+^_D_ = 5·10^18^ cm^−3^). The width of Al_0.4_Ga_0.6_As buffer layer with higher doping (N^+^_A_ = 5·10^18^ cm^−3^) placed in between GaAs and the silver substrate is 50 nm. The surface of the pore is coated with a layer of neutral Al_0.4_Ga_0.6_As of 10 nm width and the rest of the pore is filled with glass (n = 1.45). The p-n junction is formed in the middle of the cell (magenta plane), with equal doping above and below: N_A_ = N_D_ = 10^18^ cm^−3^.

Our detailed photonic crystal GaAs solar cell design is depicted in Fig. 1. Here, we show one unit cell of the square lattice of slanted conical nano-pores in GaAs with the equivalent bulk thickness of 200 nm, filled (encapsulated) with glass (n = 1.45) and placed on silver back-reflector. The height of GaAs between the contacts is 290 nm (for a 200 nm equivalent bulk thickness of GaAs). As in the case of silicon solar cells^7^, slanted conical pores provide more effective solar absorption than their non-slanted counterparts. The optimal geometrical parameters, for maximal absorption are^8^: a = 550 nm, r = 300 nm, where *a* is the lattice constant and *r* is the cone base (top) radius. To calculate the absorption (1) and the generation profile (2), we perform FDTD simulations following the algorithm described above. The maximum achievable photo-current density is given by: 
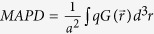
, where *a* is the lattice constant, and integration is done over the entire volume of GaAs in a single unit cell. In the case that all light from radiative recombination is assumed to escape the structure, we achieve a MAPD of 27.6 mA/cm^2^ in the 300–865 nm wavelength range, which corresponds to 88% of absorption of incoming solar power in the specified range. In this calculation, the surface of each pore is passivated with a thin layer of Al_0.4_Ga_0.6_As of 10 nm width, and the cones are filled with glass (n = 1.45). We also use a 50 nm thick buffer layer of Al_0.4_Ga_0.6_As with higher doping (N^+^_A_ = N^+^_D_ = 5·10^18^ cm^−3^) between the silver substrate and GaAs to deflect the minority carriers and reduce contact recombination. A similar 10 nm thick buffer layer with higher doping is used for the same purpose on the top of the cell covered with transparent (n = 1.9) indium tin oxide (ITO) contact with the thickness of 10 nm (see Fig. 1). A p-n junction is formed in the middle of the cell (magenta plane), with equal doping on either side: N_A_ = N_D_ = 10^18^ cm^−3^. We also vary the position of p-n junction and find that it affects the final power conversion efficiency only slightly, since the diffusion length in crystalline GaAs is more than one hundred times longer than the typical carrier transport distance in our structure. Most carriers reach the contacts without non-radiative recombination in the volume. Solar power conversion efficiency in our structure is limited by surface and contact recombination.

## Power Conversion Efficiency

**Figure 2:**
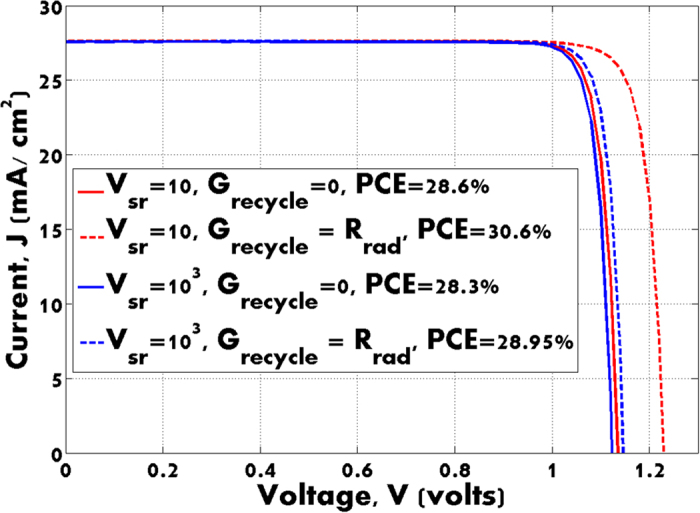
The I–V curves of the solar cell shown in Fig. 2. Solid (no photon recycling) and dash (perfect photon recycling) lines represent the same case with equal surface recombination. Blue curves correspond to the case when surface recombination velocity V_sr_ = 10 cm/s, and red curves when surface non-radiative recombination velocity V_sr_ = 10^3^ cm/s. Power conversion efficiency is denoted as ‘PCE’.

The results of solving the coupled Maxwell and drift-diffusion equations for the structure are shown in Fig. 2. The I–V characteristics for surface non-radiative recombination velocities of V_sr_ = 10^*3*^* *cm/s and V_sr_ = 10 cm/s are shown in Fig. 2. In both cases the insulating surface and contact recombination velocities are set equal. For V_sr_ = 10^*3*^* *cm/s the power conversion efficiency is 28.3%, whereas for V_sr_ = 10 cm/s, we achieve 28.6% efficiency. In the above cases, all light generated by radiative recombination was assumed to escape the structure. In the opposite limit that all radiative recombination is recycled (R_rad_ = G_recycle_), 0.7% additive efficiency increase is achieved in the first case (it reaches 29%), whereas 2% is achieved in the second case (30.6% is achieved).

The enhanced role of photon recycling with the suppression of non-radiative decay provides significant increase in V_*oc*_. The open circuit voltage behavior in Fig. 2, can be understood from the simple 1D diode equation^34^ for net current flow: *I* = *I*_*sc*_ − *I*_*sat*_[exp(*qV/kT*)−1]. Here, *I*_*sc*_ is the short-circuit photocurrent (when V = 0), V is the voltage across the contacts, k is Boltzman’s constant, the temperature T = 300 K and *I*_*sat*_ is a saturation current, determined by carrier recombination. The open circuit voltage V_oc_ is obtained by setting I = 0:


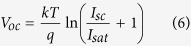


The saturation current^34^ in our case, arises from surface nonradiative recombination and bulk radiative recombination: *I*_*sat*_ = *I*_*surf*_ + *I*_*rad*_. A rough estimate of the surface recombination current, based on dimensional considerations is given by: 
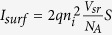
, where V_sr_ is the surface recombination velocity and S = 0.565 μm^2^ is the combined area of the GaAs – AlGaAs and GaAs – contact surfaces in each unit cell. This gives *I*_*surf*_  = 6.5·10^−19^*mA* for V_sr_ = 10^*3*^* *cm/s. Likewise, the radiative recombination current, in the absence of photon recycling is estimated by integration of the radiative recombination coefficient R_rad_ (5) over the unit cell volume: 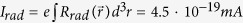. In the case of perfect photon recycling, we set *I*_*sat*_ = *I*_*surf*_. When V_sr_ = 10 cm/s, equation (6) yields V_oc1_ = 1.18 V with no photon recycling and V_oc2_ = 1.29 V with perfect photon recycling. On the other hand, when V_sr_ = 10^3^ cm/s, equation (6) yields V_oc3_ = 1.15 V with no photon recycling and V_oc4_ = 1.17 V for perfect photon recycling.

The crude model above overestimates the actual open circuit voltage in our system, but provides a simple physical interpretation of the enhanced role of photon recycling with low surface recombination velocity. In our numerical simulations (see Fig. 2), we find the corresponding voltages: V_oc1_ = 1.14 V, V_oc2_ = 1.23 V for V_sr_ = 10 cm/s and V_oc3_ = 1.12 V, V_oc4_ = 1.15 V for V_sr_ = 10^*3*^* *cm/s. Clearly, the ratios V_oc1_/V_oc2_ = 0.91 and V_oc3_/V_oc4_ = 0.98 estimated from (6) are close to those (V_oc1_/V_oc2_ = 0.92 and V_oc3_/V_oc4_ = 0.97) of our numerical simulation results in Fig. 2. This underscores the value of improved surface passivation in photonic crystal solar cells to enable the strong synergy between light-trapping and photon recycling.

Setting G_recycle_ = R_rad_ is equivalent to 100% collection of photons emitted by radiative recombination. In reality this effect is slightly less pronounced, since some of the regenerated photons escape the solar cell. We estimate the amount of re-cycled light by FDTD simulation of spatially distributed point dipoles with oscillation amplitude determined by the semiconductor drift-diffusion equations. The re-radiation strength of each dipole is chosen such that the number of photons per unit frequency interval near ω, per unit volume near 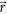 is given by:


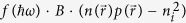
. The distribution of dipole oscillation frequencies 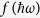 is governed by the thermal distribution of electrons and holes at the operating voltage of the solar cell^35^. For each radiating dipole at position 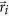 within the active region, we calculate the escaping Poynting vector flux, 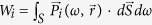, where S is a surface surrounding the entire solar cell and 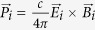 is the Poynting vector field of the i-th dipole. The total emitted radiation from the i^th^ dipole is: 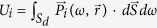, where S_d_ is a surface surrounding the dipole. S_d_ consists of a small box with faces 10 nm from the i^th^ dipole within the active region. The radiation from each dipole depends strongly on the local EM density of states. This is automatically recaptured by our FDTD algorithm. The average amount of radiation re-absorbed by the solar cell is: 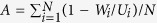, where N is the number of dipoles. As a representative simulation, we uniformly spread N = 121 dipoles throughout the active region of a vertical plane bisecting the slanted cone. For radiative recombination in this representative plane, we find that 70% of the emitted light is recycled. A more detailed description of photon recycling using a self-consistent iterative solution of the coupled Maxwell and drift-diffusion equations will be presented elsewhere.

**Figure 3:**
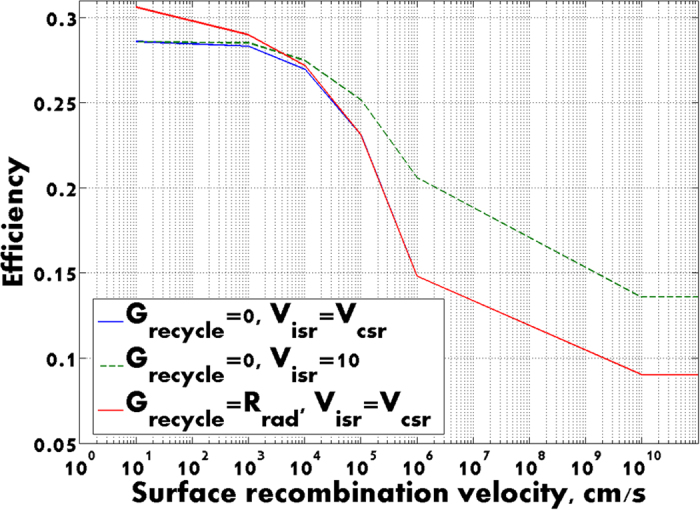
Solar cell efficiency as a function of surface recombination velocities. Blue curve depicts the efficiency when surface and contact recombination velocities are equal and there is no photon recycling. Green curve represents efficiency vs. contact recombination velocity with surface recombination velocity fixed at 10 cm/s. The red curve depicts efficiency with perfect photon recycling (G_recycle_ = R_rad_) with equal surface and contact recombination rates.

In Fig. 3 we present the dependence of power conversion efficiency on surface non-radiative recombination velocity. The blue curve illustrates the drop in efficiency with V_sr_ in the absence of photon recycling when both surface and contact recombination are set equal. This suggests that V_sr_ = 10^4^ cm/s is the tipping point, below which solar cell performance is very good but above which performance drops dramatically^24^,^26^. We attain 28.3% power conversion (no recycling) for V_sr_ = 10^3^ cm/s with Voc = 1.12 V and J_SC_ = 27.6 mA/cm^2^. These values are already very close to the current world record for single-junction GaAs solar cell efficiency without solar concentration. On the other hand, our photonic crystal solar cell uses 15 times less volume of GaAs compared to the record-setting solar cell.

The red curve in Fig. 3 depicts efficiency improvement with the perfect photon recycling. As before we set G_recycle_ = R_rad_. The most pronounced effect is achieved (see also Fig. 2), when surface and contact recombination velocities are minimal (V_sr_ = 10 cm/s). The additive contribution to solar cell performance is 2% for V_sr_ = 10 cm/s, 0.7% for V_sr_ = 10^3^ cm/s and becomes negligible for V_sr_ > 10^5^ cm/s.

Finally, we isolate the impact of contact recombination on solar cell performance (dotted green curve). In this simulation, the insulating surface recombination velocity is fixed at V_isr_ = 10 cm/s, while the contact recombination velocity V_csr_ is varied. It is seen that below 10^4^ cm/s, blue and green curves almost coincide. The difference becomes more pronounced for V_csr_ > 10^5^ cm/s. In this case, contact recombination losses alone degrades solar cell performance irrespective of the quality of passivation of all insulating surfaces.

## Conclusion

We have identified opportunities for reducing the volume of GaAs by more than an order of magnitude relative to conventional solar cells while increasing power conversion efficiencies beyond the current world record. The critical trade-off in our photonic crystal architectures is between wave-interference-based light-trapping and non-radiative carrier recombination at surfaces. The large surface area of nanostructured photonic crystals makes them prone to non-radiative losses while at the same time providing strong scattering and trapping of sunlight. As a consequence of this trade-off, we identify roughly 200 nm equivalent bulk thickness of GaAs as an ideal photonic crystal solar cell. For lower volumes of GaAs, solar absorption decreases rapidly. For larger volumes of GaAs, only slight increase in power conversion efficiency is possible. This estimate is based on a surface non-radiative recombination velocity of 10^3^ cm/s. Recent advances^27^ in surface passivation of complex silicon surfaces have provided recombination velocities on the order of 10 cm/s. If a similar degree of passivation is realized for GaAs, a 2% additive increase in power conversion efficiency is possible from the strong luminescence properties of GaAs. Our photonic crystal architecture not only traps incoming sunlight but it also facilitates trapping and re-absorption of light produced by radiative recombination of charge carriers. The effect of photon recycling is most prominent when non-radiative recombination is strongly suppressed, enabling our 200 nm GaAs solar cell to reach power conversion efficiency as high as 30.6%. This theoretical prediction is substantially above the world – record at one-sun incident flux, for a single-junction GaAs solar cell of any thickness.

## Additional Information

**How to cite this article**: Eyderman, S. and John, S. Light-trapping and recycling for extraordinary power conversion in ultra-thin gallium-arsenide solar cells. *Sci. Rep.* **6**, 28303; doi: 10.1038/srep28303 (2016).

## Supplementary Material

Supplementary Information
