# Supplementary material for: Light-trapping and recycling for extraordinary power conversion in ultra-thin gallium-arsenide solar cells
Source: Sci Rep. 2016 Jun 23;6:28303. doi: 10.1038/srep28303 (PMC4917830; doi:10.1038/srep28303)
Supplement: Supplementary Information [file srep28303-s1.doc]

**Supplementary Information**

**Light-trapping and recycling for extraordinary power conversion in ultra-thin gallium**-**arsenide solar cells**

Sergey Eyderman*, Sajeev John

^1^Department of Physics, University of Toronto, 60 St. George Street, Toronto, Ontario, M5S 1A7, Canada.

*Corresponding author e-mail: [sergey.eyderman@utoronto.ca](mailto:sergey.eyderman@utoronto.ca)

So far we considered an ultra-thin GaAs solar cell with an equivalent bulk thickness of only 200nm. It is tempting to imagine that increasing the volume of GaAs would lead to a significant increase in power conversion efficiency. Our 200nm GaAs photonic crystal provides MAPD of 27.5 mA/cm^2^ compared to the limiting photocurrent density of 30.5 mA/cm^2^ for 100% solar absorption in the 300 – 865nm wavelength range. As it turns out, the gains in MAPD by increasing the volume of GaAs are mitigated by a drop in V_oc_ caused by further recombination losses. We consider (similar to Fig.1) the variable thickness architecture shown in the inset of Fig.S1. In this new architecture, the distance between the bottom tip of the cone and Al_0.4_Ga_0.6_As buffer layer is H=50nm, with the p-n junction located in the middle (25nm below the cone tip). We keep *H* constant, while the cone height, *h*, is varied. In Fig.S1 the dependence of MAPD in the spectral range [300-865nm] on the total thickness (*h+H*) of the structure is shown. As the thickness of slanted pore PhC is increased to 1100nm the MAPD reaches J=29.8 mA/cm^2^.

We also considered the case when the cone height, *h*, is kept constant at 300nm, while the thickness *H*, of the solid part is varied. In this case, the MAPD reaches only J=28.4mA/cm^2^ (when overall thickness H+h=1100nm), showing that this choice of the architecture suffers from reduced antireflection properties relative to the deeper cone structure. Therefore, we focus on the former case with fixed H=50nm and variable cone height h.


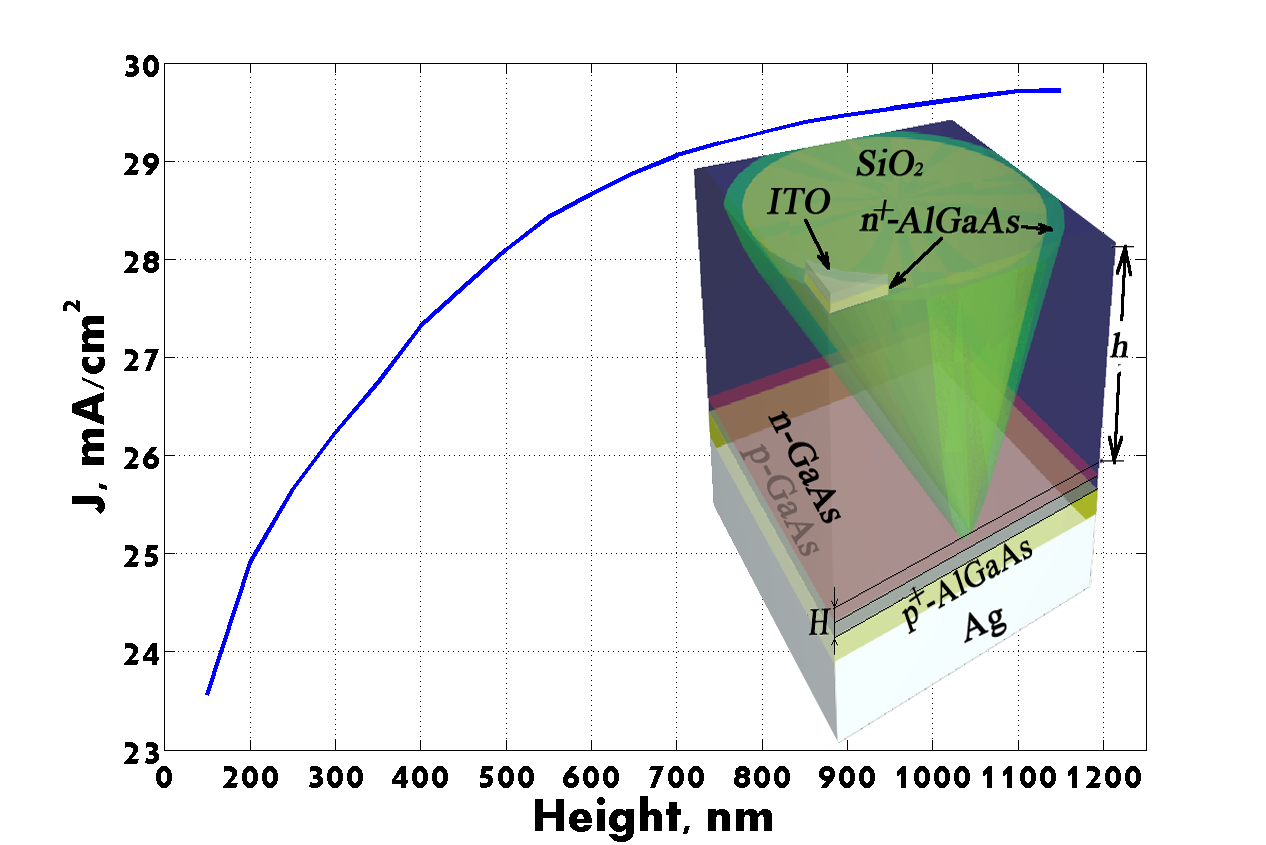


**Fig.S1** MAPD (normal incidence of AM 1.5 sunlight) dependence on the height of the inverted conical pores in GaAs, arranged in a square lattice passivated with Al_0.4_Ga_0.6_As and filled with glass. The structure is placed on silver back-reflector. The distance between the tip of the cone and Al_0.4_Ga_0.6_As buffer layer is fixed at H=50nm.The p-n junction is located in the middle (25nm below the cone tip). Both contact and surface recombinations are equal: V_isr_= V_csr_ =10^3^ cm/s. All other parameters are the same as in Fig.1. MAPD of 29.8 mA/cm^2^ for overall thickness (*h+H*) of 1.1μm is achieved.

In order to determine the efficiency dependence on the cone depth (with fixed H=50nm), we calculate four carrier generation profiles using (2), corresponding to h+H = 300, 500, 700 and 1100nm thicknesses. Solving the drift-diffusion equations (3) for each structure we find the power conversion efficiencies, shown in Fig.S2.
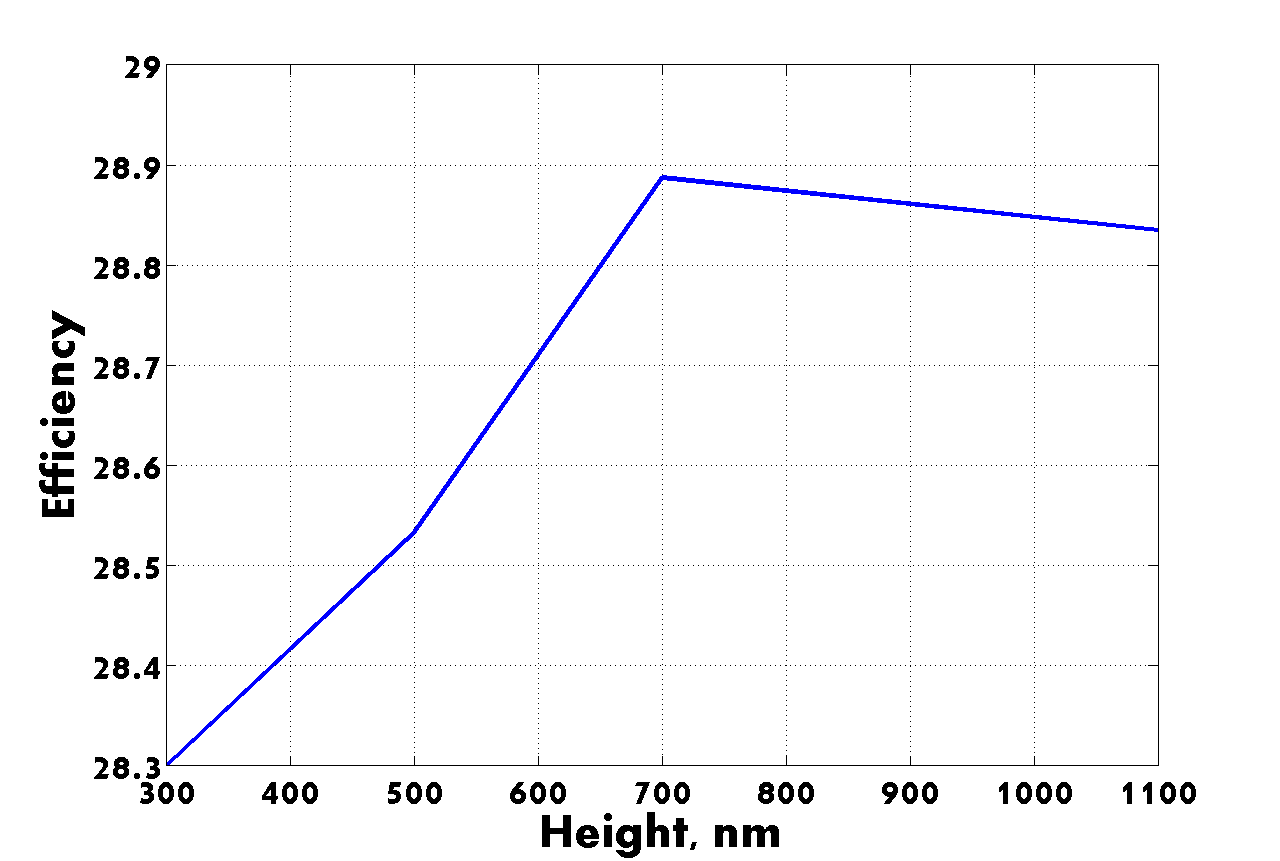


**Fig.S2** Dependence of power conversion efficiency on solar cell thickness (h+H) for the structure shown in the inset of Fig.S1. Here V_sr_ = 10^3^cm/s.

Remarkably the maximal efficiency of 28.9% is achieved at h+H=700nm thickness (450nm equivalent bulk thickness), even though the MAPD continues to grow slowly as the thickness is increased. In order to understand this behavior we plot the open circuit voltage dependence on cell thickness (see Fig.S3). V_oc_ decreases with the cell thickness due to the increase in surface area. For V_sr_ =10^3^cm/s, this increase in non-radiative surface recombination overwhelms the MAPD growth from 700 to 1100nm thickness. As a result, the power conversion efficiency reaches a maximum for a thickness of 700nm and decreases thereafter.

However, the absolute efficiency increase from 300nm height to 700nm is only 0.6%. A more dramatic increase in efficiency from 24% to 28.3% occurs between the heights of 200 nm and 300 nm. Accordingly, we suggest approximately 200nm equivalent bulk thickness (290 nm distance between contacts) as the optimal balance between power conversion efficiency and volume of GaAs required.

We also esimate the role of photon recycling, by setting G_recycle_=R_rad_. Solving the drift-diffusion equations, we attain 29.7% power conversion efficiency, for V_sr_ =10^3^cm/s, using 450nm equivalent bulk thickness of gallium arsenide. It is clear from this analysis that there is greater gain from reducing non-radiative recombination at all surfaces with only 200nm equivalent bulk thickness of GaAs than from simply using a larger volume of GaAs.


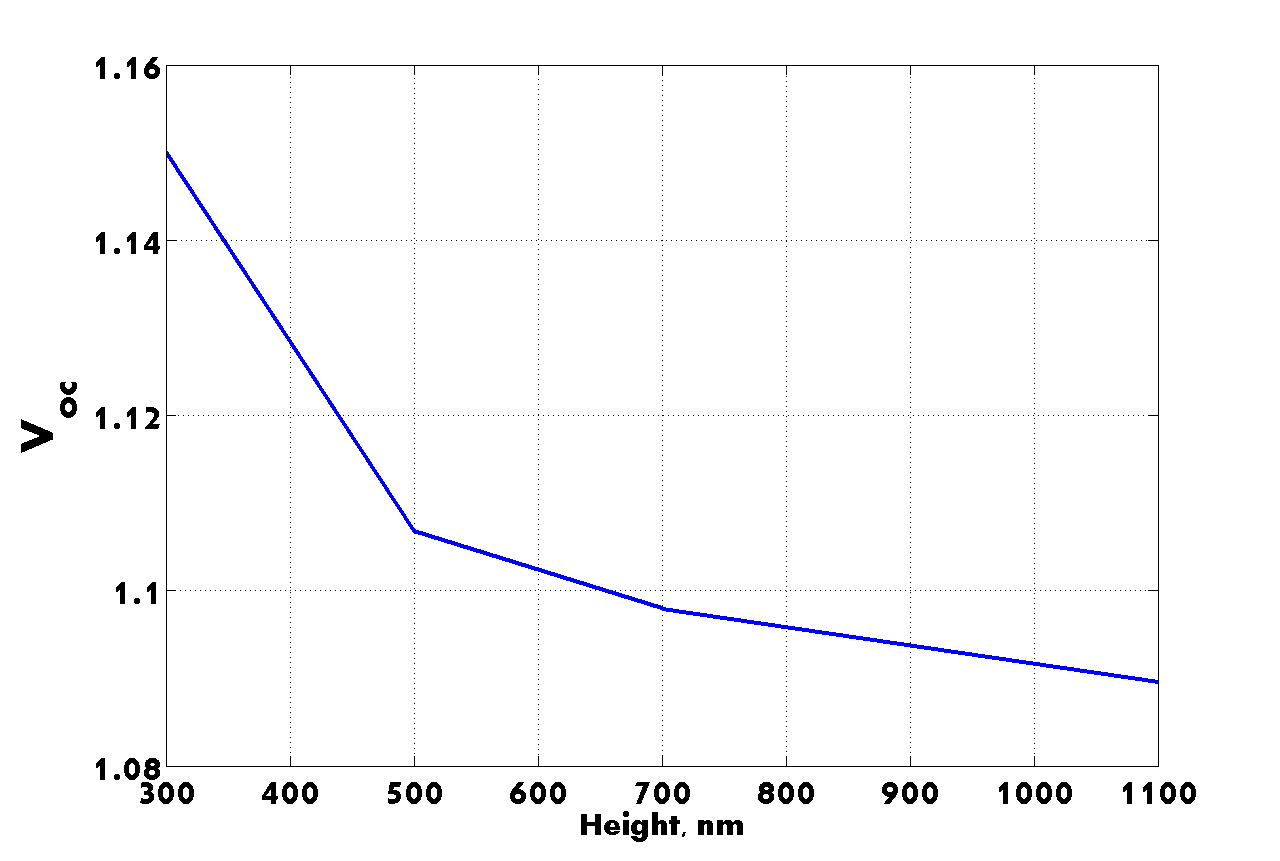


**Fig.S3** Open circuit voltage dependence on solar cell thickness (h+H) for the structure shown in the inset of Fig.S1. Here V_sr_ =10^3^cm/s.
